# (*E*)-Ethyl *N*′-(2-hydroxy­benzyl­idene)­hydrazinecarboxyl­ate

**DOI:** 10.1107/S1600536808022307

**Published:** 2008-07-19

**Authors:** Bo Gao

**Affiliations:** a Marine College, Zhejiang Institute of Communications, Hangzhou 311112, People’s Republic of China

## Abstract

There are two mol­ecules in the asymmetric unit of the title compound, C_10_H_12_N_2_O_3_, with identical conformations. Each independent mol­ecule is approximately planar and adopts a *trans* configuration with respect to the C=N double bond. Intra­molecular O—H⋯N hydrogen bonds are observed in both mol­ecules. The mol­ecules are linked into a ribbon-like structure running along the *b* axis by inter­molecular N—H⋯O and C—H⋯O hydrogen bonds. The ribbons are arranged into layers parallel to (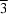
02).

## Related literature

For the properties of benzaldehyde hydrazone derivatives, see: Parashar *et al.* (1988 ▶); Hadjoudis *et al.* (1987 ▶); Borg *et al.* (1999 ▶). For a related structure, see: Shang *et al.* (2007 ▶).
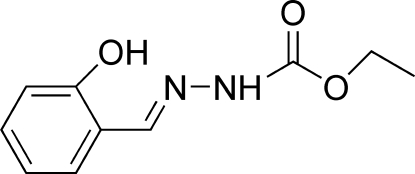


## Experimental

### 

#### Crystal data

C_10_H_12_N_2_O_3_
*M* *_r_* = 208.12
Monoclinic, 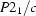

*a* = 11.535 (11) Å
*b* = 22.05 (2) Å
*c* = 9.005 (9) Å
β = 111.669 (14)°
*V* = 2129 (4) Å^3^
*Z* = 8
Mo *K*α radiation
μ = 0.10 mm^−1^
*T* = 273 (2) K
0.27 × 0.24 × 0.23 mm

#### Data collection

Bruker SMART CCD area-detector diffractometer
Absorption correction: multi-scan (*SADABS*; Bruker, 2002 ▶) *T*_min_ = 0.973, *T*_max_ = 0.978
14192 measured reflections
3713 independent reflections
1445 reflections with *I* > 2σ(*I*)
*R*_int_ = 0.146

#### Refinement

*R*[*F* ^2^ > 2σ(*F* ^2^)] = 0.058
*wR*(*F* ^2^) = 0.150
*S* = 0.78
3713 reflections
274 parameters
H-atom parameters constrained
Δρ_max_ = 0.29 e Å^−3^
Δρ_min_ = −0.16 e Å^−3^

### 

Data collection: *SMART* (Bruker, 2002 ▶); cell refinement: *SAINT* (Bruker, 2002 ▶); data reduction: *SAINT*; program(s) used to solve structure: *SHELXS97* (Sheldrick, 2008 ▶); program(s) used to refine structure: *SHELXL97* (Sheldrick, 2008 ▶); molecular graphics: *SHELXTL* (Sheldrick, 2008 ▶); software used to prepare material for publication: *SHELXTL*.

## Supplementary Material

Crystal structure: contains datablocks I, global. DOI: 10.1107/S1600536808022307/ci2635sup1.cif

Structure factors: contains datablocks I. DOI: 10.1107/S1600536808022307/ci2635Isup2.hkl

Additional supplementary materials:  crystallographic information; 3D view; checkCIF report

## Figures and Tables

**Table 1:** Hydrogen-bond geometry (Å, °)

*D*—H⋯*A*	*D*—H	H⋯*A*	*D*⋯*A*	*D*—H⋯*A*
O1—H1⋯N1	0.82	1.85	2.573 (3)	147
N2—H2*A*⋯O4^i^	0.86	2.13	2.979 (4)	170
O4—H4*A*⋯N3	0.82	1.86	2.586 (3)	146
N4—H6⋯O2^ii^	0.86	2.08	2.931 (4)	170
C5—H5⋯O5^i^	0.95	2.27	3.184 (4)	161
C15—H15⋯O1^ii^	0.95	2.46	3.371 (5)	161

Symmetry codes: (i) 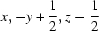; (ii) 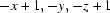.

**Fig. 1.**
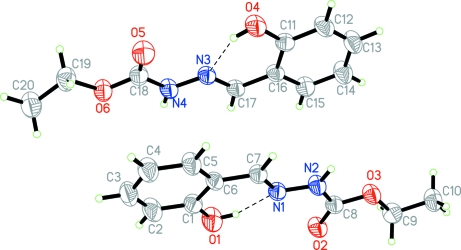
The asymmetric unit of the title compound. Displacement ellipsoids are drawn at the 30% probability level. O—H···N hydrogen bonds are shown as dashed lines.

**Fig. 2.**
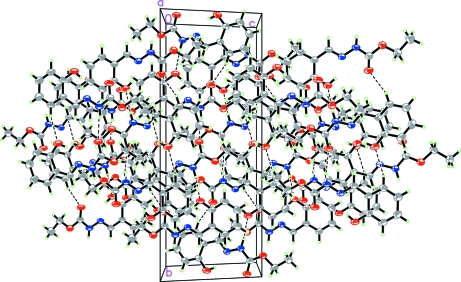
The crystal packing of the title compound, viewed approximately down the a axis. Dashed lines indicate hydrogen bonds.

## supplementary crystallographic information

### Comment

Benzaldehydehydrazone derivatives have received considerable attention for a long time due to their pharmacological activity (Parashar *et al.*, 1988) and photochromic properties (Hadjoudis *et al.*, 1987). They are important intermediates for 1,3,4-oxadiazoles, which have been reported to be versatile compounds with many properties (Borg *et al.*, 1999). We report here the crystal structure of the title compound.

The asymmetric unit of the title compound contains two independent but essentially identical molecules (Fig. 1). The corresponding bond lengths and angles of the two independent molecules agree with each other and are comparable to those observed for N'-(4-methoxybenzylidene)methoxyformohydrazide (Shang *et al.*, 2007). Each independent molecule is approximately planar and adopts a *trans* configuration with respect to the C═N bond. The dihedral angle between C1-C6 and O2/O3/N1/N2/C7-C10 planes is 7.6 (1)° and that between C11-C16 and O5/O6/N3/N4/C17-C20 planes is 5.5 (1)°. In both independent molecules intramolecular O—H···N hydrogen bonds are observed.

The molecules are linked into a ribbon-like structure running along the *b* axis by intermolecular N—H···O and C—H···O hydrogen bonds. (Fig.2). The ribbons are arranged into layers parallel to the (3 0 2) plane.

### Experimental

2-Hydroxybenzaldehyde (1.22 g, 0.01 mol) and ethyl hydrazinecarboxylate (1.04 g, 0.01 mol) were dissolved in methanol (25 ml) and left for 3 h at room temperature. The resulting solid was filtered off and recrystallized from ethanol to give the title compound in 85% yield. Single crystals suitable for X-ray analysis were obtained by slow evaporation of a methanol solution at room temperature (m.p. 455–457 K).

### Refinement

H atoms were positioned geometrically (N-H = 0.86 Å and C-H = 0.95-0.99 Å) and refined using a riding model, with *U*_iso_(H) = 1.2–1.5*U*_eq_(C).

### Crystal data

**Table:**

C_10_H_12_N_2_O_3_	*F*_000_ = 880
*M**_r_* = 208.12	*D*_x_ = 1.299 Mg m^−^^3^
Monoclinic, *P*2_1_/*c*	Mo *K*α radiation λ = 0.71073 Å
Hall symbol: -P 2ybc	Cell parameters from 3713 reflections
*a* = 11.535 (11) Å	θ = 1.9–25.0º
*b* = 22.05 (2) Å	µ = 0.10 mm^−^^1^
*c* = 9.005 (9) Å	*T* = 273 (2) K
β = 111.669 (14)º	Block, colourless
*V* = 2129 (4) Å^3^	0.27 × 0.24 × 0.23 mm
*Z* = 8	

### Data collection

**Table:**

Bruker SMART CCD area-detector diffractometer	3713 independent reflections
Radiation source: fine-focus sealed tube	1445 reflections with *I* > 2σ(*I*)
Monochromator: graphite	*R*_int_ = 0.146
*T* = 273(2) K	θ_max_ = 25.0º
φ and ω scans	θ_min_ = 1.9º
Absorption correction: multi-scan(SADABS; Bruker, 2002)	*h* = −13→12
*T*_min_ = 0.973, *T*_max_ = 0.978	*k* = −26→25
14192 measured reflections	*l* = −10→10

### Refinement

**Table:**

Refinement on *F*^2^	Hydrogen site location: inferred from neighbouring sites
Least-squares matrix: full	H-atom parameters constrained
*R*[*F*^2^ > 2σ(*F*^2^)] = 0.058	*w* = 1/[σ^2^(*F*_o_^2^) + (0.0618*P*)^2^] where *P* = (*F*_o_^2^ + 2*F*_c_^2^)/3
*wR*(*F*^2^) = 0.150	(Δ/σ)_max_ = 0.001
*S* = 0.78	Δρ_max_ = 0.30 e Å^−^^3^
3713 reflections	Δρ_min_ = −0.16 e Å^−^^3^
274 parameters	Extinction correction: SHELXL97 (Sheldrick, 2008), Fc^*^=kFc[1+0.001xFc^2^λ^3^/sin(2θ)]^-1/4^
Primary atom site location: structure-invariant direct methods	Extinction coefficient: 0.0017 (5)
Secondary atom site location: difference Fourier map	

### Special details

**Table:**

Geometry. All e.s.d.'s (except the e.s.d. in the dihedral angle between two l.s. planes) are estimated using the full covariance matrix. The cell e.s.d.'s are taken into account individually in the estimation of e.s.d.'s in distances, angles and torsion angles; correlations between e.s.d.'s in cell parameters are only used when they are defined by crystal symmetry. An approximate (isotropic) treatment of cell e.s.d.'s is used for estimating e.s.d.'s involving l.s. planes.
Refinement. Refinement of *F*^2^ against ALL reflections. The weighted *R*-factor *wR* and goodness of fit *S* are based on *F*^2^, conventional *R*-factors *R* are based on *F*, with *F* set to zero for negative *F*^2^. The threshold expression of *F*^2^ > σ(*F*^2^) is used only for calculating *R*-factors(gt) *etc*. and is not relevant to the choice of reflections for refinement. *R*-factors based on *F*^2^ are statistically about twice as large as those based on *F*, and *R*- factors based on ALL data will be even larger.

### Fractional atomic coordinates and isotropic or equivalent isotropic displacement parameters (Å^2^)

**Table:**

	*x*	*y*	*z*	*U*_iso_*/*U*_eq_	
O1	0.6761 (2)	−0.00597 (8)	0.5564 (3)	0.1012 (8)	
H1	0.6249	0.0071	0.4725	0.121*	
O2	0.43571 (18)	−0.01273 (8)	0.1327 (2)	0.0799 (7)	
O3	0.32487 (19)	0.06261 (8)	−0.0314 (2)	0.0781 (7)	
N1	0.5648 (2)	0.07322 (10)	0.3410 (3)	0.0661 (7)	
N2	0.4749 (2)	0.08742 (10)	0.1969 (3)	0.0740 (8)	
H2A	0.4578	0.1246	0.1679	0.089*	
C1	0.7458 (3)	0.04071 (13)	0.6420 (4)	0.0744 (10)	
C2	0.8384 (3)	0.02856 (15)	0.7861 (4)	0.0988 (12)	
H2	0.8519	−0.0121	0.8237	0.119*	
C3	0.9126 (3)	0.07430 (16)	0.8778 (4)	0.1005 (12)	
H3	0.9765	0.0650	0.9775	0.121*	
C4	0.8937 (3)	0.13398 (15)	0.8240 (4)	0.0933 (11)	
H4	0.9443	0.1658	0.8859	0.112*	
C5	0.8007 (3)	0.14600 (14)	0.6797 (4)	0.0804 (10)	
H5	0.7871	0.1868	0.6438	0.096*	
C6	0.7250 (3)	0.10084 (12)	0.5834 (3)	0.0635 (8)	
C7	0.6293 (3)	0.11571 (12)	0.4328 (3)	0.0669 (9)	
H7A	0.6135	0.1569	0.4007	0.080*	
C8	0.4133 (3)	0.04080 (14)	0.1007 (4)	0.0676 (9)	
C9	0.2552 (3)	0.01839 (14)	−0.1492 (4)	0.0839 (11)	
H9A	0.2072	−0.0083	−0.1044	0.101*	
H9B	0.3129	−0.0071	−0.1809	0.101*	
C10	0.1687 (3)	0.05187 (14)	−0.2907 (4)	0.0954 (11)	
H10A	0.1209	0.0228	−0.3728	0.143*	
H10B	0.2171	0.0783	−0.3338	0.143*	
H10C	0.1113	0.0765	−0.2584	0.143*	
O4	0.44788 (19)	0.28071 (8)	0.6198 (2)	0.0858 (7)	
H4A	0.4920	0.2570	0.6873	0.103*	
O5	0.7029 (2)	0.23103 (9)	0.9891 (3)	0.0972 (8)	
O6	0.75313 (19)	0.14080 (8)	1.1180 (2)	0.0784 (7)	
N3	0.5222 (2)	0.17646 (9)	0.7524 (3)	0.0661 (7)	
N4	0.5985 (2)	0.14460 (10)	0.8822 (3)	0.0722 (8)	
H6	0.5909	0.1061	0.8907	0.087*	
C11	0.3641 (3)	0.24868 (13)	0.4965 (4)	0.0665 (9)	
C12	0.2807 (3)	0.28054 (13)	0.3700 (4)	0.0814 (10)	
H12	0.2822	0.3236	0.3710	0.098*	
C13	0.1953 (3)	0.25032 (15)	0.2422 (4)	0.0898 (11)	
H13	0.1383	0.2728	0.1561	0.108*	
C14	0.1915 (3)	0.18688 (15)	0.2380 (4)	0.0929 (11)	
H14	0.1337	0.1660	0.1492	0.111*	
C15	0.2741 (3)	0.15531 (14)	0.3664 (4)	0.0786 (10)	
H15	0.2715	0.1122	0.3647	0.094*	
C16	0.3610 (3)	0.18441 (12)	0.4981 (3)	0.0623 (8)	
C17	0.4436 (3)	0.14927 (13)	0.6327 (3)	0.0638 (8)	
H17	0.4389	0.1063	0.6311	0.077*	
C18	0.6864 (3)	0.17732 (15)	0.9965 (4)	0.0728 (9)	
C19	0.8531 (3)	0.17077 (14)	1.2461 (4)	0.0906 (11)	
H19A	0.8180	0.1976	1.3072	0.109*	
H19B	0.9037	0.1958	1.2014	0.109*	
C20	0.9322 (3)	0.12260 (14)	1.3527 (4)	0.1046 (12)	
H20A	0.9974	0.1416	1.4441	0.157*	
H20B	0.9710	0.0981	1.2928	0.157*	
H20C	0.8802	0.0966	1.3909	0.157*	

### Atomic displacement parameters (Å^2^)

**Table:**

	*U*^11^	*U*^22^	*U*^33^	*U*^12^	*U*^13^	*U*^23^
O1	0.141 (2)	0.0421 (13)	0.0963 (17)	0.0008 (13)	0.0154 (15)	0.0104 (12)
O2	0.0980 (16)	0.0357 (11)	0.0895 (16)	0.0003 (11)	0.0154 (13)	0.0015 (10)
O3	0.0861 (15)	0.0492 (12)	0.0760 (15)	0.0038 (11)	0.0029 (13)	−0.0049 (11)
N1	0.0828 (18)	0.0437 (14)	0.0631 (17)	0.0054 (13)	0.0167 (15)	−0.0015 (12)
N2	0.0922 (19)	0.0389 (14)	0.0692 (18)	0.0031 (13)	0.0045 (16)	−0.0030 (13)
C1	0.097 (3)	0.049 (2)	0.070 (2)	0.0103 (18)	0.022 (2)	0.0070 (17)
C2	0.121 (3)	0.062 (2)	0.092 (3)	0.023 (2)	0.014 (3)	0.023 (2)
C3	0.103 (3)	0.093 (3)	0.078 (3)	0.023 (2)	0.001 (2)	0.012 (2)
C4	0.100 (3)	0.071 (2)	0.085 (3)	0.004 (2)	0.007 (2)	0.000 (2)
C5	0.092 (2)	0.0501 (19)	0.077 (2)	0.0031 (18)	0.005 (2)	0.0025 (17)
C6	0.073 (2)	0.0487 (18)	0.063 (2)	0.0105 (16)	0.0182 (18)	0.0068 (16)
C7	0.084 (2)	0.0419 (17)	0.065 (2)	0.0046 (16)	0.0151 (18)	−0.0006 (15)
C8	0.078 (2)	0.051 (2)	0.068 (2)	0.0029 (18)	0.0194 (19)	−0.0028 (17)
C9	0.090 (2)	0.062 (2)	0.081 (3)	−0.0088 (18)	0.009 (2)	−0.0169 (18)
C10	0.102 (3)	0.084 (2)	0.076 (2)	0.000 (2)	0.005 (2)	0.0031 (19)
O4	0.1091 (17)	0.0377 (11)	0.0897 (16)	−0.0034 (11)	0.0121 (14)	0.0022 (11)
O5	0.128 (2)	0.0383 (13)	0.0981 (17)	−0.0128 (12)	0.0095 (15)	−0.0045 (11)
O6	0.0879 (16)	0.0554 (13)	0.0721 (15)	−0.0046 (11)	0.0062 (13)	0.0023 (11)
N3	0.0805 (18)	0.0402 (14)	0.0678 (17)	0.0020 (13)	0.0159 (15)	0.0016 (13)
N4	0.0864 (19)	0.0336 (13)	0.0771 (18)	−0.0030 (13)	0.0075 (16)	0.0018 (13)
C11	0.081 (2)	0.0425 (18)	0.072 (2)	−0.0037 (16)	0.0228 (19)	−0.0017 (16)
C12	0.095 (3)	0.0512 (19)	0.089 (3)	0.0076 (19)	0.023 (2)	0.0140 (19)
C13	0.101 (3)	0.068 (2)	0.083 (3)	0.004 (2)	0.014 (2)	0.018 (2)
C14	0.099 (3)	0.070 (2)	0.088 (3)	−0.006 (2)	0.009 (2)	0.008 (2)
C15	0.091 (2)	0.0497 (18)	0.079 (2)	−0.0087 (18)	0.012 (2)	−0.0058 (18)
C16	0.070 (2)	0.0446 (17)	0.070 (2)	0.0015 (15)	0.0234 (18)	0.0022 (15)
C17	0.073 (2)	0.0402 (16)	0.072 (2)	0.0004 (16)	0.0197 (18)	0.0004 (16)
C18	0.082 (2)	0.055 (2)	0.069 (2)	0.0013 (19)	0.015 (2)	−0.0019 (18)
C19	0.097 (3)	0.075 (2)	0.079 (2)	−0.008 (2)	0.008 (2)	0.0001 (19)
C20	0.103 (3)	0.096 (3)	0.092 (3)	0.000 (2)	0.010 (2)	0.010 (2)

### Geometric parameters (Å, °)

**Table:**

O1—C1	1.358 (3)	O4—C11	1.369 (3)
O1—H1	0.82	O4—H4A	0.82
O2—C8	1.220 (3)	O5—C18	1.205 (3)
O3—C8	1.338 (3)	O6—C18	1.348 (3)
O3—C9	1.447 (3)	O6—C19	1.454 (3)
N1—C7	1.289 (3)	N3—C17	1.274 (3)
N1—N2	1.365 (3)	N3—N4	1.369 (3)
N2—C8	1.363 (3)	N4—C18	1.355 (3)
N2—H2A	0.86	N4—H6	0.86
C1—C2	1.368 (4)	C11—C12	1.381 (4)
C1—C6	1.414 (4)	C11—C16	1.418 (4)
C2—C3	1.382 (4)	C12—C13	1.379 (4)
C2—H2	0.95	C12—H12	0.95
C3—C4	1.391 (4)	C13—C14	1.400 (4)
C3—H3	0.95	C13—H13	0.95
C4—C5	1.371 (4)	C14—C15	1.384 (4)
C4—H4	0.95	C14—H14	0.95
C5—C6	1.395 (4)	C15—C16	1.395 (4)
C5—H5	0.95	C15—H15	0.95
C6—C7	1.436 (4)	C16—C17	1.458 (4)
C7—H7A	0.95	C17—H17	0.95
C9—C10	1.491 (4)	C19—C20	1.495 (4)
C9—H9A	0.99	C19—H19A	0.99
C9—H9B	0.99	C19—H19B	0.99
C10—H10A	0.98	C20—H20A	0.98
C10—H10B	0.98	C20—H20B	0.98
C10—H10C	0.98	C20—H20C	0.98
			
C1—O1—H1	109.4	C11—O4—H4A	109.4
C8—O3—C9	116.4 (2)	C18—O6—C19	114.9 (2)
C7—N1—N2	119.9 (2)	C17—N3—N4	120.9 (2)
C8—N2—N1	117.8 (2)	C18—N4—N3	116.1 (2)
C8—N2—H2A	121.2	C18—N4—H6	122.0
N1—N2—H2A	121.0	N3—N4—H6	121.9
O1—C1—C2	118.8 (3)	O4—C11—C12	118.4 (3)
O1—C1—C6	121.0 (3)	O4—C11—C16	121.4 (3)
C2—C1—C6	120.2 (3)	C12—C11—C16	120.3 (3)
C1—C2—C3	121.2 (3)	C13—C12—C11	120.5 (3)
C1—C2—H2	119.4	C13—C12—H12	119.7
C3—C2—H2	119.4	C11—C12—H12	119.7
C2—C3—C4	120.0 (3)	C12—C13—C14	120.7 (3)
C2—C3—H3	120.0	C12—C13—H13	119.6
C4—C3—H3	120.0	C14—C13—H13	119.6
C5—C4—C3	118.7 (3)	C15—C14—C13	118.4 (3)
C5—C4—H4	120.7	C15—C14—H14	120.8
C3—C4—H4	120.7	C13—C14—H14	120.8
C4—C5—C6	122.8 (3)	C14—C15—C16	122.4 (3)
C4—C5—H5	118.6	C14—C15—H15	118.8
C6—C5—H5	118.6	C16—C15—H15	118.8
C5—C6—C1	117.1 (3)	C15—C16—C11	117.6 (3)
C5—C6—C7	120.7 (3)	C15—C16—C17	120.4 (3)
C1—C6—C7	122.1 (3)	C11—C16—C17	121.9 (3)
N1—C7—C6	120.0 (3)	N3—C17—C16	119.8 (3)
N1—C7—H7A	120.0	N3—C17—H17	120.1
C6—C7—H7A	120.0	C16—C17—H17	120.1
O2—C8—O3	125.7 (3)	O5—C18—O6	125.3 (3)
O2—C8—N2	124.3 (3)	O5—C18—N4	124.8 (3)
O3—C8—N2	110.0 (3)	O6—C18—N4	110.0 (3)
O3—C9—C10	107.9 (3)	O6—C19—C20	107.7 (3)
O3—C9—H9A	110.1	O6—C19—H19A	110.2
C10—C9—H9A	110.1	C20—C19—H19A	110.2
O3—C9—H9B	110.1	O6—C19—H19B	110.2
C10—C9—H9B	110.1	C20—C19—H19B	110.2
H9A—C9—H9B	108.4	H19A—C19—H19B	108.5
C9—C10—H10A	109.5	C19—C20—H20A	109.5
C9—C10—H10B	109.5	C19—C20—H20B	109.5
H10A—C10—H10B	109.5	H20A—C20—H20B	109.5
C9—C10—H10C	109.5	C19—C20—H20C	109.5
H10A—C10—H10C	109.5	H20A—C20—H20C	109.5
H10B—C10—H10C	109.5	H20B—C20—H20C	109.5

### Hydrogen-bond geometry (Å, °)

**Table:**

*D*—H···*A*	*D*—H	H···*A*	*D*···*A*	*D*—H···*A*
O1—H1···N1	0.82	1.85	2.573 (3)	147
N2—H2A···O4^i^	0.86	2.13	2.979 (4)	170
O4—H4A···N3	0.82	1.86	2.586 (3)	146
N4—H6···O2^ii^	0.86	2.08	2.931 (4)	170
C5—H5···O5^i^	0.95	2.27	3.184 (4)	161
C15—H15···O1^ii^	0.95	2.46	3.371 (5)	161

Symmetry codes: (i) *x*, −*y*+1/2, *z*−1/2; (ii) −*x*+1, −*y*, −*z*+1.
